# Calculating Stress: From Entropy to a Thermodynamic Concept of Health and Disease

**DOI:** 10.1371/journal.pone.0146667

**Published:** 2016-01-15

**Authors:** Julie Bienertová-Vašků, Filip Zlámal, Ivo Nečesánek, David Konečný, Anna Vasku

**Affiliations:** Department of Pathological Physiology, Faculty of Medicine, Masaryk University, Kamenice 5 A18, Brno, 625 00, Czech Republic; CNRS, FRANCE

## Abstract

To date, contemporary science has lacked a satisfactory tool for the objective expression of stress. This text thus introduces a new–thermodynamically derived–approach to stress measurement, based on entropy production in time and independent of the quality or modality of a given stressor or a combination thereof. Hereto, we propose a novel model of stress response based on thermodynamic modelling of entropy production, both in the tissues/organs and in regulatory feedbacks. Stress response is expressed in our model on the basis of stress entropic load (SEL), a variable we introduced previously; the mathematical expression of SEL, provided here for the first time, now allows us to describe the various states of a living system, including differentiating between states of health and disease. The resulting calculation of stress response regardless of the type of stressor(s) in question is thus poised to become an entirely new tool for predicting the development of a living system.

## Introduction to Stress, Allostasis and Entropy

The association between chronic environmental and intrinsic factors and the pathogenesis of disease has been extensively documented throughout the history of mankind and although many attempts at characterizing the very basis of health and disease have been made, no completely satisfactory theory capable of providing an exhausting explanation of the general pathogenic processes has thus far been proposed.

One of the first explanations—functional, broadly conceived and still accepted—is Hans Selye’s General Adaptation Syndrome, a comprehensive stress theory proposed in 1936 [1], which defines stress as the “nonspecific response of the body to any demand made on it”. Although Selye was also the first to systemically address the crucial issue of the role of environmental influences in disease development, his theory did not provide a robust framework for the measurement of stress and was in effect criticized ever since the term’s introduction.

The key strength of Selye’s stress theory is also its biggest weakness: it is not per se associated with any specific mechanisms of action of individual stressors. The practical application of the theory is thus hindered by the fact that it fails to distinguish between i) internal and external causal factors, ii) physiological and pathophysiological consequences of stress-induced processes and iii) stressor-specific and non-specific response; moreover, iv) the definition of stress does not take into consideration the interpretation of the stimulus by the organism itself [2]. Dissatisfaction with *stress* as a term and with Selye’s stress theory as a whole, frequently expressed by the scientific community, is thus derived primarily from the fact that it does not allow either for the quantification of the impact of actual stressors or for any kind of evaluation of the overreaction of systems experiencing chronic stress. As a result of this, Selye’s theory is currently superseded by the novel concepts of scientific integrative medicine that integrate system biology with integrative physiology that offer more accurate and complex explanations to the biological observations. Despite of this, direct measurement/calculation of stress response still isn’t possible.

The introduction of the concept of allostasis by Sterling and Eyer in 1988 [3] constitutes an important step towards a new approach to stress. The subsequently proposed allostasis model [4], which differentiates between two types of allostatic overload, was the first to introduce an environmental component.

The concept of allostasis exhibits two key strengths: i) it provides a definition of *allostatic load* basically as the wear and tear experienced by individuals coping with repeated stressors as well as perturbations in the given system caused by environmental stimuli, and thus ii) it provides a framework for understanding changes in physiological set points and their adaptation to external stimuli over time. Although McEwen and Wingfield’s allostatic model facilitates the approximation of stress response by measuring indirect parameters associated above all with HPA axis activation, it currently provides the only applicable framework for measuring stress as such, although with substantial limitations.

The primary weakness of the allostasis concept is that allostatic markers are by their very nature highly cross-sectional and thus incapable of directly reflecting the cumulative functioning of most stressors. Moreover, since energy input and expenditure are both overly inconsistent and poorly understood to be used for measuring allostatic load, the entire theory is based only on indirect metabolic parameters. In addition, the theory largely relies on rather vague connection between metabolism and energy consumption. Hence, utility of allostasis concept for stress measurement is currently limited. A basic description of the terms we use throughout the following text is provided in Table 1.

**Table 1:** Basic terminology.

**Homeostatic stationary state**	The optimal setting which a system has a natural tendency to return to. Homeostasis may be considered to constitute oscillations around this stationary state, where the origin of the oscillations is both internal (daily/seasonal routines and basic metabolic demands) and external, i.e. coming from the environment (perturbations from the environment, demands other than daily/seasonal routines). A succession of homeostatic stationary states may be viewed as homeodynamic.
**Homeostatic state**	Any state exhibiting a natural tendency to reach a homeostatic stationary state.
**Homeostatic region**	A set of all homeostatic states adjacent to a given homeostatic stationary state.
**Allostatic verge**	The point at which a system leaves a homeostatic region and enters the process of allostasis, i.e. the point where it becomes more advantageous to change the setting of the whole system rather than to try reach the original homeostatic stationary state. In other words, the “breaking point” of the regulation.

Based on the concept of association of entropy production in living systems, introduced in 1944 in Schrödinger’s outstanding work *What is Life*? [5], it is generally accepted that an increase in order within an organism is compensated for by an increase in disorder outside this organism, mainly via the loss of heat into the environment. Although it may seem then that the dynamics of life are at odds with the second law of thermodynamics—which states that the entropy of an isolated system can only increase—this paradox is seemingly resolved by the open nature of living systems, which basically indicates that a system can exchange either heat or matter or both with its environment. In order to sustain vital processes, living organisms continuously use high-energy nutrients, generating heat and entropy via metabolic pathways and transferring entropy to the environment through various waste channels, namely perspiration and heat transfer across the skin, in order to maintain a biological system’s fixed thermal state.

As a result, the human body may be described as an open thermodynamic system. Since entropy and entropy-related variables are directional with time, it seems only logical to apply the concept of entropy to typically physiological developmental processes, i.e. growth and aging.

Generally, living systems are likely to represent extensive metabolic networks regulating cellular physiology and metabolic efficiency, both in relation to gene expression, and representing fluctuations of matter, charge, heat and information. Since the concept of entropy is based on the notion of dynamic diversity and fluctuations of microscopic processes underlying macroscopic cellular states, it is likely that the characterization of network dynamics originating from cellular robustness will constitute a more useful approach than network characterizations relying solely on topology [6]. Manke et al. [6] emphasize the importance of structural network entropy-related observables as correlates of the dynamic properties of a biological system, suggesting that some properties of large systems can be effectively described by means of a number of macroscopic parameters regardless of the concurrent ignorance of microscopic processes. Manke et al. build this presumption on the relationship between the Gibbs distribution over microstates and various macroscopic properties which can be derived from valid equilibrium states and is formally extending the Gibbs distribution to non-equilibrium systems at steady state [6], suggesting that some systemic properties can be explained without resorting to microscopic details.

The above approach could thus provide a tremendously useful concept in human physiology and pathophysiology, i.e. areas where the characterization of all molecular—cellular interactions with respect to health—disease dynamics seems to be overwhelmingly difficult.

Furthermore, it must be mentioned that it would be wrong to limit the interpretation of entropy generation in living systems solely to (1) experimental calorimetric measurements or allometric laws accounting for metabolic rates and (2) energy/entropy balances in a control volume surrounding the human body, as is the case in several existing studies dedicated to lifespan estimation models (e. g. rate of living theory, radical oxygen species theory and rate of entropy generation). These concepts generally do not account for ATP production during metabolism and their use is thus substantially limited. The use of cumulative specific entropy production for the viability prognosis of an organ/organ system/organism based on the assumption that the growth of organ and body mass along with metabolic rate may be used to calculate entropy associated with organ stress, as suggested by Annamalai et al. [7], and in view of ATP production, diet composition, diet energy content and physical activity, apparently represents a more comprehensive and viable approach [8]. Calculations employed throughout this study are based on Annamalai’s model and presumptions [7].

Although a great deal of effort has been devoted to developing a reliable model for the determination of the entropy production rate in the human body [9–11], a universally accepted entropy-based model which would explain stress-related response has yet to be introduced.

## Stress Entropic Load (SEL)

By modifying the model of rate of entropy generation of lifetime expectancy, Silva and Annamalai [10] presented the REG (MREG) theory, a concept substantially improved by accounting for ATP production and physical activity levels, and proposed an expression for estimating global entropy generation within the whole body (biological system). The authors estimated the specific entropy generation rate of the whole body σM, (J/ {kg body K}) as a function of the organism’s age and proposed an estimation of variation in specific entropy generation, i.e. SEG (J/ {K kg body}), of the whole body with age (t). To estimate the total lifespan entropy generation of the human body in terms of the entropy generation of each individual organ, the availability analysis for each isothermal organ was applied. Three main nutrient classes were considered within this model: carbohydrates (CH), fats (F) and proteins (P). The metabolic efficiency was the same as availability efficiency for an isothermal system in previous literature [12]; for any nutrient n, it was defined as:

$$\eta_{n}={\left(\frac{\Delta G_{ATP}}{\Delta G_{c}}\right)}_{n}$$

where (ΔG_c_)n is change in the Gibbs function of nutrient “n” during metabolism. The Gibbs free energy change of nutrients during metabolism—i.e. ΔG_c,n_—is a function of temperature, pressure and mole fraction and approximately equals ΔG_c,n_°–i.e. ΔG_c,n_ ≈ G_c,n_°–which implies that nutrients, oxidants, CO_2_ and H_2_O exist as pure species in reactants and products [7].

The model we are about to present uses the presumptions of Silva et Annamalai and generally relies on two prerequisites: i) the overall changes in the entropy of an open system, expressed as:

$$dS_{TOT}=dS_{PROD}+dS_{FLOW}$$

where *dS*_*FLOW*_ denotes changes in entropy corresponding to the flow of energy and mass across system boundaries and where *dS*_*PROD*_ denotes changes in entropy corresponding to processes taking place inside the system (entropy production), and ii) the second law of thermodynamics, which states that *dS*_*PROD*_ ≥ 0.

Based on these universally valid concepts, we presume that *dS*_*PROD*_ includes a base component (*dS*_*BASAL*_), i.e. the intrinsic increase of system complexity independent of its surroundings—associated with growth, development and ageing, and a reactive component (*dS*_SEL_), i.e. an increase in entropy due to a wide range of environmental influences which reflects the energy cost of adaptation:

$$dS_{PROD}=dS_{BASAL}+dS_{SEL}$$

In order to make individual rates of change of system entropy comparable, it is necessary to introduce the concept of specific rate of change of system entropy per unit mass (σ(t)), i.e. proceed from the presumption that the amount of entropy produced is mass-dependent:

$$\sigma \left(t\right)=\frac{Ś\left(t\right)}{m\left(t\right)}$$

[13,14]

By integrating σ_SEL_, it is possible to acquire the cumulative specific change of entropy, which we define as **stress entropic load** (s_SEL_):

$$s_{SEL}\left(t\right):=\int_{t_{conc}}^{t}\sigma_{SEL}\left(\tau \right)d\tau$$ (1)

Where SEL is defined as an integral from the time of conception of a given individual to a later point in time of the specific rate of change of the entropy σSELof the individual.

The above is the key theoretical expression of our entropy-based stress mode.

## Stress Entropic Load Change

The theoretical expression established above allows us to derive an increase in SEL for a reasonable and practically applicable time interval [t_1_, t_2_], calculating it as the difference between stress entropic load values at the extreme points of the interval:

$$\Delta_{\left[t_{1},t_{2}\right]}s_{SEL}:=s_{SEL}\left(t_{2}\right)-s_{SEL}\left(t_{1}\right)=\int_{t_{1}}^{t_{2}}\sigma_{SEL}\left(\tau \right)d\tau ,$$ (2)

Where *t*_2_ ≥ *t*_1_ ≥ *t*_*conc*_

In case the given interval is clearly delineated, the following abbreviated equation may be employed:

$$\Delta s_{SEL}=s_{SEL}\left(t_{2}\right)-s_{SEL}\left(t_{1}\right).$$

Within a given interval [t_1_, t_2_], SEL change in produced entropy is calculated as the difference between the overall change of entropy and change of entropy due to mass and energy flows across system boundaries (all values per unit of mass):

$$\Delta s_{PROD}=\Delta s_{TOT}-\Delta s_{FLOW},$$ (3)

Where The following increases–Δ*s*_*TOT*_, Δ*s*_*FLOW*_ and Δ*s*_*BASAL*_—are defined analogously to Δ*s*_*PROD*_.

SEL has been defined as part of entropy production, i.e. the difference between total entropy production and entropy production associated with basal metabolic function. It therefore follows that

$$\Delta s_{SEL}=\Delta s_{PROD}-\Delta s_{BASAL}.$$ (4)

As long as the system is not subject to stress (i.e. in case Δ*S*_*SEL*_ = O), then Δ*S*_*BASAL*_ = Δ*S*_*PROD*_, i.e. an increase in entropy caused by basal metabolic function is directly equal to an increase in entropy production.

The above may be summed up as

$$\begin{array}{ll} \Delta s_{SEL} & =\Delta s_{PROD}-\Delta s_{BASAL}\\ & =\Delta s_{PROD_{\left(stressed\right)}}-\Delta s_{PROD_{\left(no\;stress\right)}}\\ & ={\left[\Delta s_{TOT}-\Delta s_{FLOW}\right]}_{\left(stressed\right)}-{\left[\Delta s_{TOT}-\Delta s_{FLOW}\right]}_{\left(no\;stress\right)}. \end{array}$$ (5)

(Fig 1)

**Fig 1:**
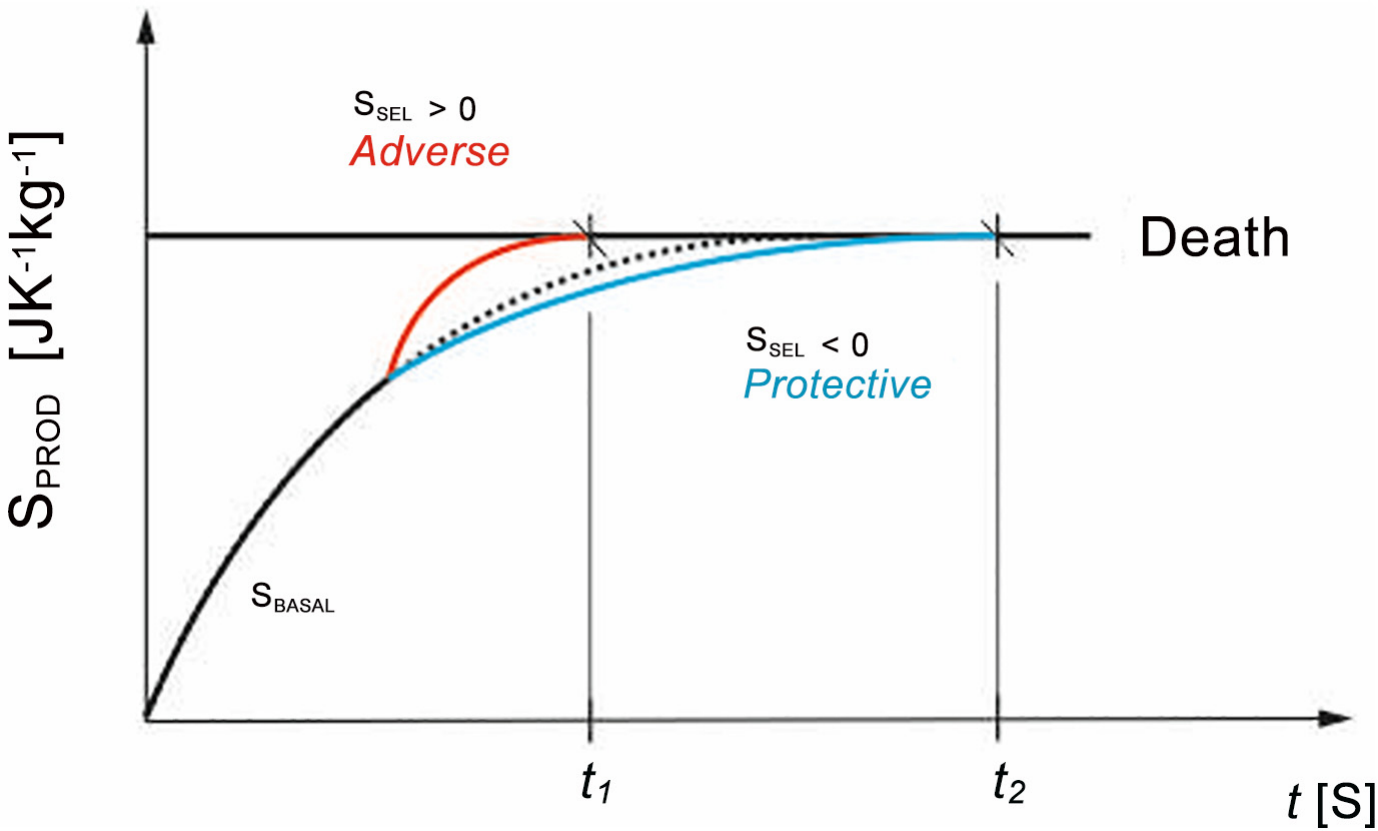
Δs_PROD_ = Δs_BASAL_+Δs_SEL_. Graphic representation of basal and SEL-associated entropy production and their relation to system death. t—time, S_prod_—production of entropy, S_SEL_—stress entropic load

The first element [Δ*s*_*TOT*_−Δ*s*_*FLOW*_]_(*stressed*)_ represents the total amount of produced entropy of a system in a given time interval, i.e. what is actually measured in an individual, while the second element [Δ*s*_*TOT*_−Δ*s*_*FLOW*_]_(*no stress*)_ represents the basic function of an individual's metabolism, which must be estimated (as it cannot be directly or indirectly measured, since the individual may be subject to measurement only once during the interval [t_1_, t_2_]; moreover, he/she will have been subject to various stressors during the course of the measurement).

A specific measurement of entropy production during the interval [t_1_, t_2_] thus helps establish the value of [Δ*s*_*TOT*_−Δ*s*_*FLOW*_]_(*stressed*)_, (ideally) by means of establishing rates *σ*_*TOT*_(*t*) and *σ*_*FLOW*_(*t*) at time *t*, *t*_1_ ≤ *t ≤ t*_2_, subtracting and integrating them, i.e.

$${\left[\Delta s_{TOT}-\Delta s_{FLOW}\right]}_{\left(stressed\right)}=\int_{t_{1}}^{t_{2}}(\sigma_{TOT}\left(t\right)-\sigma_{FLOW}\left(t\right))dt=\int_{t_{1}}^{t_{2}}\sigma_{PROD_{\left(stressed\right)}}\left(t\right)dt.$$ (6)

In practice, measurements should be carried out so that the chosen sequence of time points divides the interval [t1, t2] in a sufficiently “fine” manner, so as to facilitate subsequent measurements of additional variables.

The second parameter in Eq (6) is expressed analogously as

$$\begin{array}{ll} {\left[\Delta s_{TOT}-\Delta s_{FLOW}\right]}_{\left(no\;stress\right)} & =\int_{t_{1}}^{t_{2}}(\sigma_{TOT_{\left(no\;stress\right)}}\left(t\right)-\sigma_{FLOW_{\left(no\;stress\right)}}\left(t\right))dt\\ & =\int_{t_{1}}^{t_{2}}\sigma_{PROD_{\left(no\;stress\right)}}\left(t\right)dt. \end{array}$$ (7)

Two methods may be employed to establish $\sigma_{PROD_{\left(no\;stress\right)}}\left(t\right)$. It may be modeled, i.e. by determining in advance how the variable will develop over time; alternatively, the following approximation may be used: as long as the interval [t_1_, t_2_] is “sufficiently short”, i.e. the rate of entropy production associated with basal metabolic function remains unchanged throughout this interval (e.g. when the duration measured is much shorter than the average lifespan of an individual), then

$${\left[\Delta s_{TOT}-\Delta s_{FLOW}\right]}_{\left(no\;stress\right)}=\underset{const.}{\underset{}{\underset{︸}{\sigma_{PROD\left(no\;stress\right)}}}\left(t\right).\left(t_{2}-t_{1}\right)}$$ (8)

(this approximation is necessary in order to indicate that the above difference may be considered constant for the duration of a relatively short period of time during which the measurement is performed).

## Calculating $\sigma_{PROD_{\left(stressed\right)}}\left(t\right)$

According to the above, it follows that $\sigma_{PROD_{\left(stressed\right)}}\left(t\right)=\frac{{\dot{s}}_{PROD}\left(t\right)}{m\left(t\right)}$. The following shall all take place in time *t*; in order to simplify, the variable *t* is omitted from the subsequent calculations. Next, ${\dot{S}}_{PROD}$ must be expressed in terms of measurable variables, described and clarified below.

Entropy production rate is calculated as

$${\dot{S}}_{PROD}={\dot{S}}_{TOT}-{\dot{S}}_{FLOW},$$ (9)

where

$${\dot{S}}_{TOT}=\frac{{\dot{Q}}_{p}-{\dot{Q}}_{e}}{T_{body}},$$ (10)

and

$${\dot{S}}_{FLOW}={\dot{S}}_{FLOW_{\left(mass\right)}}-{\dot{S}}_{FLOW_{\left(energy\right)}}.$$ (11)

${\dot{S}}_{FLOW_{\left(mass\right)}}$ and ${\dot{S}}_{FLOW_{\left(energy\right)}}$ constitute the rates of change of entropy due to the flow of mass or energy, respectively, across system boundaries.

The two variables may be expressed as follows:

$${\dot{S}}_{FLOW_{\left(mass\right)}}=\dot{\sigma }\left(O_{2}\right)M\left(O_{2}\right)-\dot{\sigma }\left(CO_{2}\right)M\left(CO_{2}\right)+\dot{\sigma }\left(H_{2}O\right)M\left(H_{2}O_{in}\right)-\dot{\sigma }\left(H_{2}O\right)M\left(H_{2}O_{out}\right)+\;\sigma \left(O_{2}\right)\dot{M}\left(O_{2}\right)-\sigma \left(CO_{2}\right)\dot{M}\left(CO_{2}\right)+\sigma \left(H_{2}O\right)\dot{M}\left(H_{2}O_{in}\right)-\sigma \left(H_{2}O\right)\dot{M}\left(H_{2}O_{out}\right)$$ (12)

(as the molar entropy content of O, CO and HO varies with temperature, which varies with time, a time-dependent concept of molar entropy contents may be proposed; if the molar entropy content of these substances remains constant, then calculating ${\dot{S}}_{FLOW_{\left(mass\right)}}$ by means of Eq (13) will only require parameters listed on the second row) and

$${\dot{S}}_{FLOW_{\left(energy\right)}}={\dot{S}}_{in}-({\dot{S}}_{out}+{\dot{S}}_{env}+{\dot{S}}_{evp}+{\dot{S}}_{rad}+{\dot{S}}_{env_{res}}+{\dot{S}}_{evp\_res}),$$ (13)

where

$$\begin{array}{c} {\dot{S}}_{in}=\frac{4}{3}\eta A\sigma_{SB}T_{air}^{3},\\ {\dot{S}}_{out}=\frac{4}{3}\epsilon A\sigma_{SB}T_{skin}^{3},\\ {\dot{S}}_{env}=\frac{{\dot{Q}}_{env}}{T_{skin}},\\ {\dot{S}}_{evp}=\frac{{\dot{Q}}_{evp}}{T_{body}},\\ {\dot{S}}_{rad}=\frac{{\dot{Q}}_{rad}}{T_{skin}},\\ {\dot{S}}_{env\_res}=\frac{{\dot{Q}}_{env\_res}}{T_{skin}},\\ {\dot{S}}_{evp\_res}=\frac{{\dot{Q}}_{evp\_res}}{T_{body}}. \end{array}$$ (14)

Combining the above equations facilitates the calculation of the stress entropic load change of an individual during the time interval [t_1_, t_2_], expressed as

$$\begin{array}{ll} \Delta s_{SEL}= & \int_{t_{1}}^{t_{2}}[\frac{{\dot{Q}}_{p}-{\dot{Q}}_{e}}{T_{body}}-\dot{\sigma }\left(O_{2}\right)M\left(O_{2}\right)+\dot{\sigma }\left(CO_{2}\right)M\left(CO_{2}\right)-\dot{\sigma }\left(H_{2}O\right)M\left(H_{2}O_{in}\right)\\ & +\dot{\sigma }\left(H_{2}O\right)M\left(H_{2}O_{out}\right)-\sigma \left(O_{2}\right)\dot{M}\left(O_{2}\right)+\sigma \left(CO_{2}\right)\dot{M}\left(CO_{2}\right)\\ & -\sigma \left(H_{2}O\right)\dot{M}\left(H_{2}O_{in}\right)+\sigma \left(H_{2}O\right)\dot{M}\left(H_{2}O_{out}\right)-\frac{4}{3}\eta A\sigma_{SB}T_{air}^{3}\\ & +\frac{4}{3}\epsilon A\sigma_{SB}T_{skin}^{3}+\frac{{\dot{Q}}_{cnv}}{T_{skin}}+\frac{{\dot{Q}}_{evp}}{T_{body}}+\frac{{\dot{Q}}_{rad}}{T_{skin}}+\frac{{\dot{Q}}_{cnv\_res}}{T_{skin}}+\frac{{\dot{Q}}_{evp\_res}}{T_{body}}]\frac{1}{m\left(t\right)}dt\\ & -\overset{t_{2}}{\underset{t_{1}}{\int }}\sigma_{PROD_{\left(no\;stress\right)}}\left(t\right)dt. \end{array}$$ (15)

An overview of variables used in the model is provided in Table 2.

**Table 2:** Variables description for the model.

Variable	Unit	Description
*A*	m^2^	area of human body
*M*(CO_2_)	mol	amount of CO_2_ liberation
*M*(H_2_O_*in*_)	mol	amount of H_2_O uptake
*M*(H_2_O_*out*_)	mol	amount of H_2_O liberation
*M*(O_2_)	mol	amount of O_2_ uptake
$\dot{M}{\text{(CO}}_{2}\text{)}$	mol⋅s^−1^	CO_2_ liberation rate
$\dot{M}{\text{(H}}_{2}{\text{O}}_{in}\text{)}$	mol⋅s^−1^	H_2_O uptake rate
$\dot{M}{\text{(H}}_{2}{\text{O}}_{out}\text{)}$	mol⋅s^−1^	H_2_O liberation rate
$\dot{M}{\text{(O}}_{2}\text{)}$	mol⋅s^−1^	O_2_ uptake rate
${\dot{S}}_{PROD}$	J⋅s^−1^K^−1^	entropy production rate of a human body
${\dot{S}}_{TOT}$	J⋅s^−1^K^−1^	change in entropy content in the human body
${\dot{S}}_{FLOW}$	J⋅s^−1^K^−1^	net entropy rate flow into body due to energy and mass exchange
${\dot{S}}_{FLOW\left(nass\right)}$	J⋅s^−1^K^−1^	net entropy rate flow into body due mass exchange
${\dot{S}}_{FLOW\left(energy\right)}$	J⋅s^−1^K^−1^	net entropy rate flow into body due energy exchange
*${\dot{S}}_{cmv}$*	J⋅s^−1^K^−1^	entropy production rate loss by convection
*${\dot{S}}_{evp}$*	J⋅s^−1^K^−1^	entropy production rate loss by evaporation
*${\dot{S}}_{rad}$*	J⋅s^−1^K^−1^	entropy production rate loss by radiation
*${\dot{S}}_{cnv\_res}$*	J⋅s^−1^K^−1^	entropy production rate loss by respiratory convection
*${\dot{S}}_{evp\_res}$*	J⋅s^−1^K^−1^	entropy production rate loss by respiratory evaporation
σ_*PROD*_	J⋅s^−1^K^−1^	specific entropy production rate from a human body
σ_*TOT*_	J⋅s^−1^K^−1^	specific change in entropy content in the body
σ_*FLOW*_	J⋅s^−1^K^−1^	specific net entropy rate flow into body due to energy and mass exchange
σ(CO_2_)	J⋅K^−1^mol^−1^	entropy content of CO_2_ liberation
σ(H_2_O)	J⋅K^−1^mol^−1^	entropy content of H_2_O liberation rate
σ (O_2_)	J⋅K^−1^mol^−1^	entropy content of O_2_ uptake
$\dot{\sigma }\left(CO_{2}\right)$	J⋅K^−1^mol^−1^s^−1^	entropy content of CO_2_ liberation rate
$\dot{\sigma }\left(H_{2}O\right)$	J⋅K^−1^mol^−1^s^−1^	entropy content of H_2_O liberation rate
$\dot{\sigma }\left(O_{2}\right)$	J⋅K^−1^mol^−1^s^−1^	entropy content of O_2_ uptake rate
*${\dot{Q}}_{p}$*	J⋅s^−1^	heat produced in the body
*${\dot{Q}}_{e}$*	J⋅s^−1^	heat eliminated from the body
_*${\dot{Q}}_{cnv}$*_	J⋅s^−1^	convective heat loss rate
*${\dot{Q}}_{evp}$*	J⋅s^−1^	evaporative heat loss rate
*${\dot{Q}}_{rad}$*	J⋅s^−1^	radiation heat loss rate
*${\dot{Q}}_{cnv\_res}$*	J⋅s^−1^	respiratory convective heat loss rate
*${\dot{Q}}_{evp\_res}$*	J⋅s^−1^	respiratory evaporative heat loss rate
*T_air_*	K	air temperature
*T_skin_*	K	skin temperature
*T_body_*	K	body temperature
σ*_SB_*	5,67.10^−8^ J⋅m^−2^s^−1^K^−4^	Stefan—Boltzmann constant
ε		emissivity of human skin for infrared radiation
η		absorbity of human skin for infrared radiation

This equation allows us to use measurable quantities in order to calculate the stress burden exerted on a given system. Calculating SEL change therefore enables us to introduce a novel entropy-based model of health and disease.

## Stress Entropic Load and Regulatory Feedbacks

The preceding section introduces a concept of entropy generation and accumulation in biological mass (preferentially in tissues and organs). Nevertheless, it must be mentioned that previous attempts have successfully applied these principles on the subcellular and cellular level [6,15]; e.g. Manke et al. suggest that knockouts of proteins with a large contribution to network entropy are preferentially lethal, while West et al. propose that by integrating gene expression data with a protein interaction network it is possible to demonstrate that cancer cells are characterized by an increase in network entropy. As examples of system failure in the case of intact organs have empirically been recorded, it cannot be simply stated that physical entropy accummulation is the only factor which predicts system (organism) adaptation failure. In other words, it is necessary to simultaneously address the question of entropy generation in regulatory feedbacks as well.

In most accepted concepts, stress is by definition linked to HPA axis activation, even though there are other mechanisms of stress-related activation such as the sympathoadrenomedullar axis.

For the sake of simplification and better comprehension of our concept, let us consider the single model of the HPA axis as the single—and thus crucial—element activated during stress response. The HPA axis, including its hippocampal components, has been previously characterized as a mathematical four-element model addressing—among other things—ultradian rhythmicity and containing multiple fixed points for hypercortisolemic and hypocortisolemic depression [16]. In this mathematical model, the HPA axis is considered a dynamic system (e.g. a system of differential equations) governing CRH, ACTH and cortisol levels as time-dependent variables. Based on this model, we propose that Kolmogorov-Sinai entropy (KS entropy) may be defined for this dynamic system. It is important to note that KS entropy was previously linked to physical entropy by Latora and Baranger [17].

Based on the approach suggested by Latora, for the purpose of defining the KS entropy of the regulatory feedbacks, we use the fact that it is equal to the sum of the positive Lyapunov exponents [18]. Our definition for the out-of-equilibirum physical entropy generated in the mass is therefore as follows:

S=const−I (16)

where I is Shannon information (as previously outlined by Latora et al.). In our understanding of the term, KS entropy is a far-reaching generalization of Shannon information [19] and hence a theoretical link between KS entropy and physical entropy may be established.

In the suggested HPE axis model, we presume that the KS entropy increase due to the activation of feedback could be associated with an increase in the physical entropy of the involved tissues and vice versa. However, such an assumption does not necessarily imply that an increase in cumulative tissue entropy results in increased KS entropy. The establishment of a relationship between the KS entropy of the regulatory loop and the physical entropy of the tissues represents a huge research challenge and current results are thus far inconclusive [17].

More specifically, we propose that the such an approach may be used for the definition of the stress entropic load of a regulatory feedback loop as in the case of static tissues—namely to define dS_BASAL_ as the basic entropy production of the feedback loop and dS_SEL_ as the reactive component of the entropy production due to pertubations from the environment and/or within the body. However, the description of entropy production in the regulatory feedbacks is extensive and goes beyond the scope of this paper.

## Towards a Thermodynamic Entropy-Based Concept of Health and Disease

In this theory, we perceive stress as anything that may lead to destabilization of a system independently of the quality or modality of a given stressor. The accumulation of stress-associated entropy leads to an allostatic process, which eventually results in a state of adaptation/maladaptation once a new set point is established. As the triggering of an allostatic process constitutes a key moment in the development of possible system pathology, being able to express it mathematically is crucial.

Based on our theory, each organ/tissue can be numerically characterized by certain amount of cumulative entropy at each time point that may predict future failure of the system. However, in accordance with the theory of Goldstein et al. [20], it is not only the “wear and tear” of the tissue themselves that predicts system failure, but rather the “wear and tear” of the regulatory feedbacks involved. In principle, the dynamic aspects of our theory (entropy production associated with regulatory feedbacks) is based on two presumptions: 1) that the KS entropy can be defined for all the regulatory feedbacks at all their hierarchical levels (as these feedbacks could defined by a system of differential equations and thus can be considered dynamical systems) and 2) Latora et al’s [17] suggestion that KS entropy is connected to physical entropy in the mass (i.e. in this case tissue/organs).

Using this as a departure point, we propose that entropy of the regulatory feedback loop is inevitably bound with physical entropy of the tissues involved (which does not exclude the possibility of the loop failure with intact tissues with low cumulative entropy, as observed in the clinical practice in extreme stressors). It can be also speculated that the amount of KS entropy of the loop relates to the efficiency of the particular feedback loop and that there is a certain threshold of cumulative KS entropy of the loop that predicts the transition from the negative feedback to the positive feedback regulation. To give a concrete biological example, in the setting of critical illness, there is a strong irregularity of the whole process of HPA axis regulation that subsequently translates into “independence” of ACTH and cortisol secretion and the level of the cross-approximate entropy characterizing this independence can be used for estimation of patient prognosis [21]. Based on the proposed theory, this can be explained in the following way—due to the stressing event, the reactive component of the entropy production in the loop increased rapidly and reached the threshold value for transition from one state into another. As a result of this, the feedback changed its behavior (e.g. decrement of efficiency of the negative regulation and further transition towards positive vicious cycle of concurrent cortisol and ACTH increase). Hence, we propose that using our concept, it may become possible to numerically characterize the “point of no return” of the regulatory feedback (as the critical value of cumulative KS entropy of the loop) as a certain critical amount of stress entropy production.

Thus far, the boundary between homeostasis and allostasis has evaded mathematical expression; by calculating SEL change, our model makes such an expression possible. The establishment of such a model requires the introduction of several key terms, explained in the following passage.

**Fig 2:**
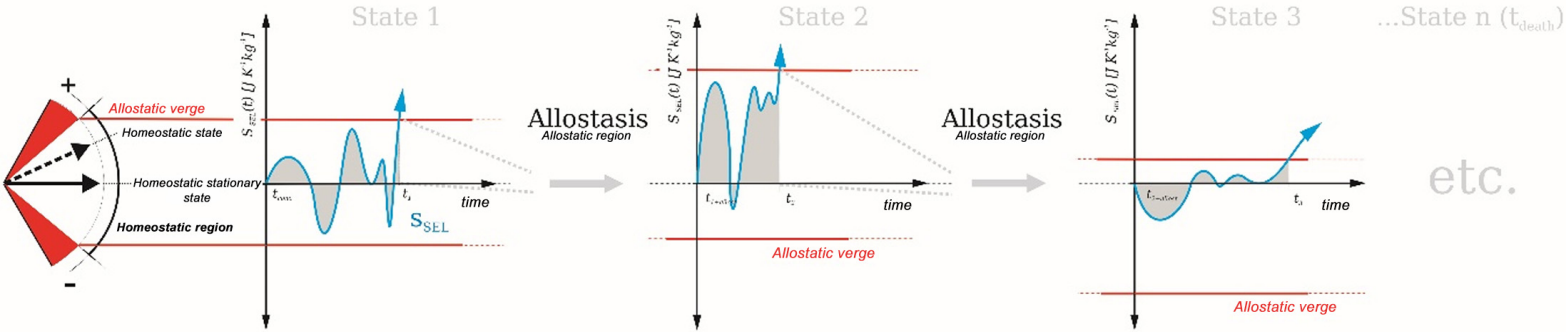
The lifetime of a living system as a repeated/successive transformation from one homeostatic stationary state (with its own homeostatic region) to another. t = time, t_1_, t_2_, t_3_ = separate time points, t_conc_ = time of conception (i.e. zygote creation), t_1+allost_ = allostatic process at time point t1, t_2+allost_ = allostatic process at time point t2, S_SEL_—stress entropic load.

Any state of a given system (organism) within a given homeostatic region exhibiting a natural tendency to reach a homeostatic stationary state is considered here a homeostatic state. For the purpose of our model, homeostatic states are determined by the influence of external stressors and/or internal errors in the system’s feedback loops. Once their influence reaches critical severity, determined by the cumulative nature of SEL at a given point in time, the system crosses the boundary of a homeostatic region. The establishment of SEL change values allows us to precisely locate this boundary, which we define as an allostatic verge. Once the value of SEL change in a given setting surpasses the value of the allostatic verge, it enters an allostatic region where the process of allostasis eventually culminates in the setting of a new homeostatic region associated with a new homeostatic stationary state. This process is repeated over and over in time (Fig 2) and may thus be labeled as homeodynamic [22].

Since the allostatic verge may be indicative of transformations of the system, including e.g. the emergence of disease, the ability to mathematically express it is a big step towards the general prediction of states of health and disease.

## Conclusion

Using entropy as a departure point, we have arrived at a holistic thermodynamic model of health and disease, whose universal character incidentally corresponds to Selye’s general theory of stress but doesn’t contradict the current understanding of stress. Our model makes it possible to calculate stress, i.e. quantify its accumulation based on the cumulative production of entropy associated with a given stressor or a combination of stressors in a specific individual and/or in a specific regulatory feedback loop in a given time interval, independently of the character of the stressor(s) in question. SEL-derived allostatic verges may be used for the prediction of transitions from one state of health or disease to another—both in an individual and in a population—and may thus constitute an immensely useful tool for the prediction of the stability of biological systems.

Since our model contains elements which may all be measured in clinical practice, e.g. in ICU, we expect that its future application will have extensive ramifications for patients and even populations. While we will continue to explore the possibilities opened up by this research, we encourage other research groups to test and validate our theoretical findings.
